# Efficacy of Carraguard®-Based Microbicides *In Vivo* Despite Variable *In Vitro* Activity

**DOI:** 10.1371/journal.pone.0003162

**Published:** 2008-09-08

**Authors:** Stuart G. Turville, Meropi Aravantinou, Todd Miller, Jessica Kenney, Aaron Teitelbaum, Lieyu Hu, Anne Chudolij, Tom M. Zydowsky, Michael Piatak, Julian W. Bess, Jeffrey D. Lifson, James Blanchard, Agegnehu Gettie, Melissa Robbiani

**Affiliations:** 1 Center for Biomedical Research, HIV and AIDS Program, Population Council, New York, New York, United States of America; 2 AIDS and Cancer Virus Program, SAIC-Frederick, Inc., National Cancer Institute, Frederick, Maryland, United States of America; 3 Tulane National Primate Research Center, Tulane University Health Sciences Center, Covington, Louisiana, United States of America; 4 Aaron Diamond AIDS Research Center, Rockefeller University, New York, New York, United States of America; National Cancer Institute, United States of America

## Abstract

Anti-HIV microbicides are being investigated in clinical trials and understanding how promising strategies work, coincident with demonstrating efficacy *in vivo*, is central to advancing new generation microbicides. We evaluated Carraguard® and a new generation Carraguard-based formulation containing the non-nucleoside reverse transcriptase inhibitor (NNRTI) MIV-150 (PC-817). Since dendritic cells (DCs) are believed to be important in HIV transmission, the formulations were tested for the ability to limit DC-driven infection *in vitro* versus vaginal infection of macaques with RT-SHIV (SIVmac239 bearing HIV reverse transcriptase). Carraguard showed limited activity against cell-free and mature DC-driven RT-SHIV infections and, surprisingly, low doses of Carraguard enhanced infection. However, nanomolar amounts of MIV-150 overcame enhancement and blocked DC-transmitted infection. In contrast, Carraguard impeded infection of immature DCs coincident with DC maturation. Despite this variable activity *in vitro*, Carraguard and PC-817 prevented vaginal transmission of RT-SHIV when applied 30 min prior to challenge. PC-817 appeared no more effective than Carraguard *in vivo*, due to the limited activity of a single dose of MIV-150 and the dominant barrier effect of Carraguard. However, 3 doses of MIV-150 in placebo gel at and around challenge limited vaginal infection, demonstrating the potential activity of a topically applied NNRTI. These data demonstrate discordant observations when comparing *in vitro* and *in vivo* efficacy of Carraguard-based microbicides, highlighting the difficulties in testing putative anti-viral strategies *in vitro* to predict *in vivo* activity. This work also underscores the potential of Carraguard-based formulations for the delivery of anti-viral drugs to prevent vaginal HIV infection.

## Introduction

Education and condom use can help limit HIV-1 transmission [1]–[3], but as currently applied do not represent definitive solutions. Thus, additional strategies are required to stem the spread of infection [4]. Vaccine and microbicide strategies tested to date have not been successful [5]–[8]. In light of recent setbacks in vaccine research [9], there is an even greater need to identify alternative approaches that reduce the risk of acquiring HIV-1 infection. Microbicides, operationally defined as substances intended to reduce or prevent transmission of HIV and/or other sexually transmitted infections (STIs) when applied topically to genital mucosal surfaces (www.microbicide.org), represent one such option. While the efficacy of a topically applied microbicide is likely to depend on the timely and correct application of the product relative to potential exposure, the availability of such products still represents an important strategy that could help stem the spread of HIV-1. Experience with such products may also usefully inform the development of alternative, coitus independent strategies.

Development of current and future microbicides must take into account our cumulative knowledge of HIV transmission. The early events leading to HIV passage across the genital mucosa likely involves HIV capture by a wide variety of molecules on the surface of epithelial cells and/or leukocytes followed by infection of permissive target cells within the tissues. *In vitro* virus transmission models have shown that dendritic cells (DCs) can capture virus and efficiently transmit these virions to CD4 T cells across infectious synapses between DCs and T cells [10]–[14]. CCR5-using HIV (R5 HIV) can productively infect immature DCs, like those located at the body surfaces, with the newly produced viruses being readily transferred to T cells [15]. In addition, both immature and mature DCs can effectively internalize viruses and transfer these virions to T cells, in the absence of productive infection of the DCs [10]–[12], [14], [16]–[18]. It is likely that immediate capture of virus by mucosa-associated DCs then transmits the virus to CD4 T cells, where robust virus replication occurs [10], [15], [19], consistent with other evidence for a more dominant role for CD4 T cells in the subsequent amplification of virus [20]–[22]. Thus, we postulated that the ability of candidate compounds to block infection in DCs or DC-T cell co-cultures may provide a more rigorous and relevant *in vitro* evaluation of the capacity of potential microbicide formulations.

Microbicide strategies currently being explored include broad-acting formulations that block HIV attachment (e.g., Carraguard, Pro 2000, BufferGel), specific small molecule inhibitors that target viral or cellular molecules critical for virus attachment, entry, or fusion (e.g., CCR5 inhibitors, fusion inhibitors like T-1249, anti-envelope Abs), and anti-viral drugs (e.g., PMPA, TMC-120, UC-781, MIV-150) [23]. The appeal of the broad-acting compounds is that they should prevent HIV interactions with any cell type (independent of the molecules involved) while also potentially having activity against other STIs. Carraguard, is a representative of this group, and has been shown to have potent activity against X4 HIV isolates with more limited activity against R5 HIV isolates *in vitro* [24]–[30], as well as having activity against other STIs [31]–[36]. A recently completed Phase III efficacy trial testing the ability of Carraguard to prevent HIV infection in women revealed that Carraguard is safe and while the frequency of HIV infection was lower in the Carraguard arm (134 vs 151 seroconversions in the placebo group), this was not a statistically significant difference [8]. In addition to safety, the inherent rheological properties and stability profile of Carraguard renders it a promising formulation vehicle, which may also have some anti-viral activity, to which other anti-viral agents could be added to provide a more effective microbicide.

The aim of this study was to evaluate the capacity of a microbicide (PC-817) comprised of Carraguard and the non-nucleoside reverse transcriptase inhibitor (NNRTI) MIV-150 to inhibit DC-driven infection *in vitro* and prevent vaginal immunodeficiency virus infection in macaques. MIV-150 (developed by Medivir, AB, Sweden, and licensed to the Population Council) is a tight-binding NNRTI with a rapid association and slow dissociation rate, which has activity against a variety of HIV-1 isolates, as well as HIV-2 [37]. MIV-150 also has virucidal activity against cell-free HIV and its anti-viral activity is not affected by seminal fluid [37]. We provide the first evidence of the additive effects of Carraguard and MIV-150 against DC-mediated infections *in vitro* and demonstrate the efficacy of Carraguard-based gels and topically applied MIV-150 *in vivo*.

## Materials and Methods

***Cell culture and reagents:*** SUPT1/CCR5 CL.30 cells (provided by J. Hoxie, University of Pennsylvania) and 174×CEM cells (AIDS Research and Reagent Program, courtesy of Peter Cresswell) were maintained in RPMI 1640 (Cellgro; Fisher Scientific, Springfield, N.J.) with 10% (v/v) heat-inactivated fetal calf serum (FCS, Cellgro). The TZM.bl cell line (AIDS Research and Reagent Program, courtesy of Dr. John C. Kappes, Dr. Xiaoyun Wu and Tranzyme Inc) was maintained in DMEM (Cellgro) with 10% (v/v) heat-inactivated FCS. Peripheral blood mononuclear cells (PBMCs) were isolated from HIV seronegative leukocyte-enriched preparations purchased from the New York Blood Center using Ficoll-Hypaque density gradient centrifugation (Amersham Pharmacia Biotech, Uppsala, Sweden). Monocytes were isolated using CD14 magnetic cell sorting (Miltenyi Biotec, Auburn, CA), with washing and elution in cold 1× PBS supplemented with 1% AB human serum (Cellgro) and 1 mM EDTA (Sigma). Monocyte purity was verified in each experiment by CD14 (MP9) and CD3 (Leu-4) staining (both Becton Dickinson, San Jose, CA), with cut-off purities of 2% CD3 T cells. Monocytes were subsequently cultured in RPMI 1640 (Cellgro) containing 10 mM HEPES (GIBCO-BRL, Life Technologies, Grand Island, NY), 2 mM L-glutamine (GIBCO-BRL), 50 μM 2-mercaptoethanol (Sigma, St. Louis, MO), penicillin (100 U/ml)-streptomycin (100 μg/ml) (GIBCO-BRL), and 1% heparinized human plasma (Innovative Research, Southfield, MI) supplemented with 100 U/ml recombinant human interleukin-4 (IL-4) (R&D Systems, Minneapolis, MN) and 1000 U/ml recombinant human granulocyte-macrophage colony-stimulating factor (GM-CSF) (Biosource/Invitrogen, Carlsbad, CA). To generate mature DCs, day 5-cultured immature DCs were exposed to a maturation cocktail of IL-1β (10 ng/ml), IL-6 (1,000 U/ml), TNF-α (10 ng/ml) (all from R&D Systems, Minneapolis, MI) and PGE_2_ (1 μg/ml) (Sigma) for 48 hours. The phenotype of immature and mature DCs was routinely monitored by two-color flow cytometry using FITC-conjugated mouse Ab against HLA-DR (Becton Dickinson) combined with the following panel of phycoerythrin (PE)-conjugated mouse anti-human monoclonal Abs (MAbs): anti-CD25, -CD80, -CD86 (all Becton Dickinson), and -CD83 (PN IM2218; Immunotech, Marseille, France).

***Microbicide preparations:*** Carraguard (Lot numbers 032805, 102505, 032906-A, 011005-B, and 010908) was prepared as a 3% (w/v) stock as described [34]. PC-817 (Lot numbers 032805, 102705, 040306-B, 011005, and 032707-A) was prepared adding a DMSO (Sigma) or ethanol solution of MIV-150 (Medivir AB, Sweden) to Carraguard, to a final concentration of 500 μM. 2.5% (25 mg/ml) methylcellulose (MC; Lot numbers 032805, 110205, 033006-A, 011005-A, 032807, and 011008) (Fisher) was used as a placebo vehicle control gel for the *in vivo* studies. To test the *in vivo* activity of MIV-150 alone, MIV-150 was mixed with 25 mg/ml MC (Lot numbers 040306-A, 032707B, and 011908). All gels were stored at room temperature. *In vitro* assays with MIV-150 were set up using 10 mM MIV-150 stocks dissolved in DMSO. 3% Carraguard stock solutions were diluted initially 1∶10 (v/v) with 1× PBS using a positive displacement pipette (Eppendorf, Hamburg, Germany).

***Virus stocks and titering:*** HIV_MN_ and HIV_Bal_ stocks were sucrose gradient purified lots #P3764 and #P3953 (courtesy of the AIDS and Cancer Virus Program, SAIC-Frederick, Inc., National Cancer Institute, Frederick, MD). The RT-SHIV construct is a hybrid of SIVmac239 bearing the reverse transcriptase gene derived from HIV HXB2 [38], [39]. RT-SHIV stocks for *in vivo* inoculations were grown in PHA activated human PBMCs (kindly provided by Disa Böttiger, Medivir AB, Sweden). RT-SHIV stocks were titered using the 174×CEM cell line and TCID_50_ was calculated according to the Reed and Muench formula.

For *in vitro* assays, a purified and high titer stock of RT-SHIV was generated as follows: 4 liters of viral supernatant were produced in the 174×CEM cell line and harvested over a period of 28 days. Viral supernatant was pre-cleared of cellular debris, by centrifugation at 1800×g for 30 min at 4°C using a benchtop centrifuge (Eppendorf). Virus was then concentrated 100 fold, using a Labscale tangential filter flow apparatus connected in parallel with two Pellicon XL 50 Cassettes with 1000 kDa molecular weight cut-off (Millipore, Billerica, MA). For 37 ml of virus filter concentrate, virus pellets were generated by ultracentrifugation in a SW28 rotor (Beckman-Coulter, Fullerton, CA) at 100,000 g through a 1 ml 20% glycerol cushion and then virus was resuspended in 400 μl of PBS and layered onto a 9 step 24% to 56% sucrose gradient. Virus was subsequently ultracentrifuged in a SW55Ti rotor (Beckman-Coulter) at 100,000×g for 3 hours with acceleration and deceleration set at 5 and 9 respectively. For sucrose gradients, peak viral fractions were harvested by analyzing A_280_ using a spectrophotometer and were later confirmed to correspond to peak infectivity using the TZM.bl cell line [15]. Harvested fractions were diluted 1 in 5 ml in 1× PBS and subsequently pelleted at 100,000×g for 90 min in a SW55 rotor (Beckman-Coulter). The pellets were resuspended overnight in 1 ml of PBS and stored at −80°C. The titer (2.49×10^8^ TCID_50_/ml) was determined using 174×CEM cells as described above.

***HIV/SIV infections and mature DC transfer assays:*** TZM.bl cells (plated at 5×10^3^ cells/well in 96 well flat-bottomed plates 16 hours earlier) or PBMCs activated for 48 hours with 5 μg/ml PHA (Sigma) (10^6^ cells/ml in 200 μl in 96 well round-bottomed plates) were treated with compounds for 30 min at 37°C and then challenged with 300 TCID_50_ of HIV_Bal_ or HIV_MN_ or 600 TCID_50_ of RT-SHIV. Activated PBMCs were recultured with complete media supplemented with 10 U/ml of IL2(Roche). For immature and mature DCs, 1.5×10^5^ cells (in 150 μl) were pretreated with compounds and pulsed with either 3000 TCID_50_ of HIV_Bal_, 4500 TCID_50_ of RT-SHIV or 3000 TCID_50_ of VSVg pseudotyped, delta HIV envelope NL43 in 96 well V-bottomed plates (Corning, NY). After 2 hours at 37°C, mature DCs were washed 4 times in media and then 10^3^ DCs were added to 5×10^3^ TZM.bl indicator cells or 5×10^3^ DCs were also added to equal numbers of either SUPT1/CCR5 CL.30 cells (for HIV_Bal_) or 174×CEM cells (for RT-SHIV). Detection of virus transfer to TZM.bl cells was by X-gal staining as described [15], [40]. Detection of transfer to SUPT1/CCR5 CL.30 cells was by Q-PCR for HIV gag DNA and RT-SHIV transfer to 174×CEM cells was by Q-PCR for SIV gag DNA, as a function of cell numbers by using Q-PCR for albumin DNA [41], [42]. Virus-pulsed immature DCs were washed before being cultured in 96 well round-bottomed plates at 10^6^ cells/ml in 200 μl of IL-4/GM-CSF media. Infection of immature DCs was monitored using intracellular stain for HIV gag p24 [43]. The percent inhibition of infection was calculated using the following equation:
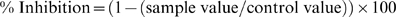



***Microbicide application and in vivo challenge:*** Adult female Chinese rhesus macaques (*Macaca mulatta*) were housed at the Tulane National Primate Research Center (TNPRC; Covington, LA). All studies were approved by the Animal Care and Use Committee of the TNPRC. Animal care procedures were in compliance with the regulations detailed in the Animal Welfare Act [44] and in the “Guide for the Care and Use of Laboratory Animals” [45]. All naïve animals tested negative for simian type D retroviruses, simian T cell leukemia virus-1, and SIV prior to use. Prior to virus challenge, animals received a single 30 mg i.m. injection of Depo-Provera. 35 days later, the macaques were sedated and 3 ml of compound were introduced atraumatically into the vaginal vault using a pliable French catheter. 1 ml of virus was applied 30 min later. At appropriate time points, pre and post viral challenge, animals were anesthetized with ketamine-HCl (10 mg/kg) prior to EDTA blood samples being taken (no more than 10 ml/kg/month/animal).

***Anti-CD8 depletion:*** Monkeys were treated with the mouse-human chimeric anti-CD8 mAb cM-T807 (NIH Nonhuman Primate Reagent Resource-Beth Israel Deaconess Medical Center, Boston, MA), receiving 10 mg/ml s.c. at day 0, followed by 5 mg/kg i.v. on days 3, 7, and 10 [46]. To verify CD8 cell depletion, whole blood was stained according to the manufacturer's guidelines for phycoerythrin (PE)-conjugated anti-CD8 (clone DK25; BD Pharmingen), fluorescein isothiocyanate (FITC)-conjugated anti-CD4 (clone L200; Dako), peridinin-chlorophyll-Cychrome (PerCP-Cy5.5)-conjugated anti-CD3 (clone SP34; BD Pharmingen).

***Plasma viral load:*** Plasma was collected from whole EDTA blood after bench top centrifugation (Eppendorf) at 800×g for 10 min. Contaminating platelets were removed by a second centrifugation at 800×g for 10 min. Plasma was then stored in 1 ml aliquots at −80°C until plasma viral load RNA detection. Measurement of plasma viral loads by quantitative RT-PCR was performed as previously described [47], [48]. We defined animals that were “infected” as those which recorded greater than 1000 RNA copies/ml in ≥2 samples post infection. Animals defined as “uninfected” had undectectable viral RNA for the duration of the viral challenge study (20 weeks) or <1000 RNA copies per ml at <2 time points post challenge.

***ELISPOT assay:*** ELISPOT assays were performed as previously described [47], [49] using 300 ng p27/ml of AT-2 inactivated SIVmneE11S [50] (Lot# p3926, courtesy of the AIDS and Cancer Virus Program, SAIC-Frederick) as the SIV antigen (vs the no virus microvesicle controls). SIV-specific responses were determined by subtracting the responses detected in control cultures from those induced by AT-2 SIV. In each experiment, PBMCs were also cultured with 5 μg/ml Concanavalin A (Sigma) to control for PBMC functionality and assay integrity. Spots were counted using an AID ELISPOT reader (Cell Technology, Columbia, MD) with once optimized settings through all experiments and the mean (±SEM) numbers of spot forming cells (SFCs) from triplicate or duplicate cultures per animal were enumerated.

***SIV specific antibody response:*** Plasma samples obtained were monitored for the presence of SIV envelope Abs by using an established ELISA protocol [51].

***Whole blood CD8/CD4 T-cell counts:*** Absolute CD4 and CD8 cell counts were monitored by TruCount (BDBiosciences, Palo Alto, CA) staining of whole blood at the indicated time points.

***Statistical analyses used in this study:*** Unless otherwise stated, data was tested for normal distribution using Origin software (Shapiro-Wilk test) (Originlab corporation, Northhampton, MA). For statistical comparisons, 2-tailed and paired *t* tests were used for the *in vitro* analyses. Fisher's Exact was calculated for *in vivo* analyses [52], with the aid of software published online at http://www.langsrud.com/fisher.htm. Standard *p* values <0.05 were taken as statistically significant.

## Results

### Carraguard can inhibit or enhance infection *in vitro*

In order to evaluate a combined MIV-150/Carraguard formulation in macaques, it was necessary to use a virus isolate that was sensitive to the drug while also infectious *in vivo*. Consistent with the lack of activity of other NNRTIs against the reverse transcriptase of SIV, there was little or no *in vitro* activity of MIV-150 against wild type SIVmac239, SHIV-162P3, or SIVmac316 (data not shown). Thus, a chimeric RT-SHIV isolate expressing HIV-1 reverse transcriptase was chosen, which has been shown to be infectious *in vivo* and sensitive to MIV-150 [38].

**Figure 1:**
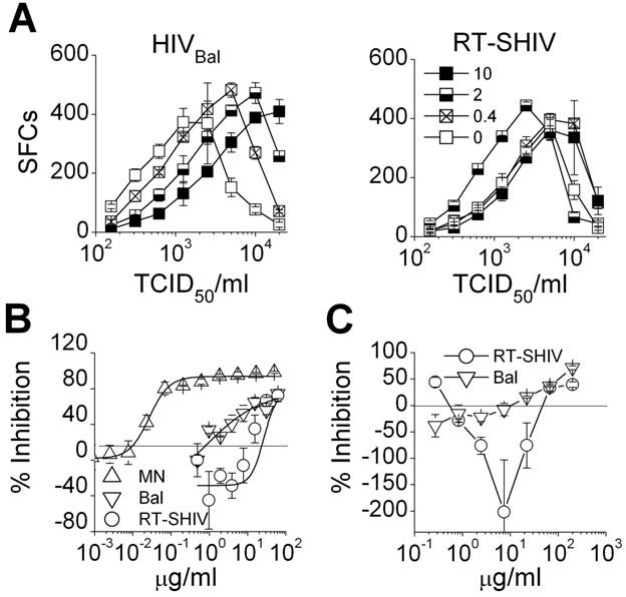
Carraguard can inhibit or enhance cell-free infection. (A) TZM.bl cells were exposed to 0–10 µg/ml of Carraguard, before graded doses of HIV_Bal_ or RT-SHIV were added. 24 h later, the media was replaced and cells were cultured for 4 d. The numbers of β-gal expressing SFCs per well are shown (mean±SD, triplicate cultures). (B) Titrated amounts of Carraguard were tested against of HIV_MN_ (MN, up triangles), HIV_Bal_ (Bal) (down triangles), or RT-SHIV (circles) in the TZM.bl cell line as in (A). The data are shown as the percent inhibition (mean±SD, triplicate cultures) of infection in the test conditions relative to the no Carraguard control. Negative % inhibition values represent enhancement, with no inhibitor effect at 0%. (C) Titrated amounts of Carraguard were added to PHA activated PBMCs before the cells were cultured with Bal or RT-SHIV for 5 d. Infection was measured by Q-PCR. Data are shown for triplicate cultures (mean±SD). Data in (A–C) are representative of 3 independent experiments with different donors in each case.

The first aim was to compare the ability of Carraguard to block RT-SHIV versus HIV infection in standard *in vitro* assay systems. Using TZM.bl cells, HIV_Bal_ was inhibited by Carraguard in dose dependent manner over the viral titration, while Carraguard was ineffective against RT-SHIV at or below 6000 TCID_50_/ml (p>0.05, Fig. 1A). Surprisingly, there was a statistically significant increase in RT-SHIV infection (300–3000 TCID_50_/ml) in the presence of very low doses (∼2 µg/ml) of Carraguard (*p*<0.05). At higher virus inocula there were reductions in infections and this correlated with loss of the cellular integrity due to extensive viral cytopathic effect (evident upon microscopic examination). Titrated amounts of Carraguard were tested against sub-saturating doses of RT-SHIV vs HIV_Bal_ and HIV_MN_ (Fig. 1B). As expected, the X4 HIV_MN_ strain was potently inhibited in a dose-dependent manner (IC_50_ = 0.030 µg/ml±0.007; n = 3). In contrast, the HIV_Bal_ inhibition curve was non sigmoidal and Carraguard was two orders of magnitude less potent against this isolate (IC_50_ = 4.17 µg/ml±2.35; n = 3). RT-SHIV was not only three orders of magnitude less sensitive to Carraguard (IC_50_ = 27.39 µg/ml±6.77; n = 3) than HIV_MN_, but at amounts of <10 µg/ml of Carraguard there was evidence of enhancement (although this was not statistically significant, *p*>0.05).

To evaluate whether this enhancement was restricted to the TZM.bl cell line, activated PBMCs were treated with varying doses of Carraguard before being challenged with HIV_Bal_ or RT-SHIV. In contrast to the TZM.bl cell line, there was significant enhancement of infection with HIV_Bal_ below 10 µg/ml of Carraguard (*p*<0.02 for 2 µg/ml Carraguard vs the media control) and the enhancement of RT-SHIV was even more pronounced with significant levels of enhancement between 2 and 20 µg/ml (*p*<0.05 over this range) (Fig. 1C).

### Carraguard enhances DC-mediated amplification of virus in T cells

Since DCs are so effective at capturing HIV [53], [54], it is likely that DC-driven dissemination and amplification of virus in CD4 T cells occurs at the mucosa *in vivo* [10], [42]. We set out to establish a robust, reproducible assay with which we could monitor putative microbicide formulations for their ability to prevent such DC-mediated virus spread. In every experiment we utilized mature DCs and compared CD4 T cell lines to the TZM.bl cells as the recipients, to control for donor specific-differences (in the recipient cells) and the data shown are representative of both co-culture methods.

**Figure 2:**
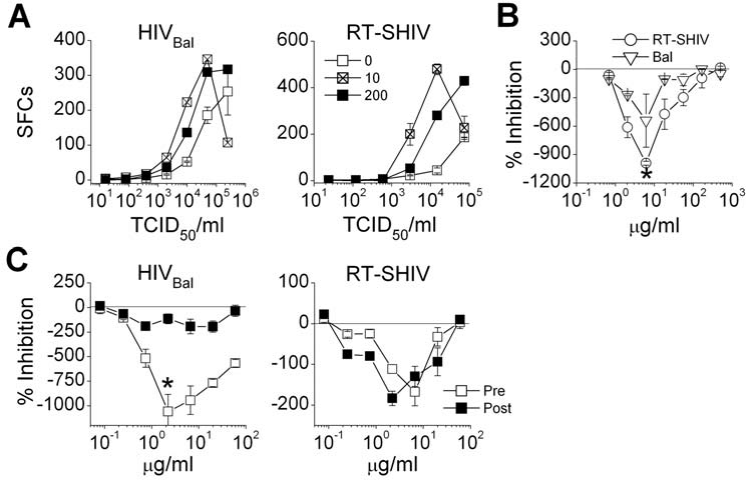
Carraguard augments mature DC-mediated amplification of infection. (A) Mature moDCs were pre-incubated with 0, 10, or 200 µg/ml Carraguard and challenged with graded doses of Bal or RT-SHIV, washed, and co-cultured with TZM.bl cells. Mean SFCs (±SD, triplicate conditions) are shown from 1 of 4 experiments. (B) Carraguard-treated mature DCs were pulsed with Bal or RT-SHIV, washed, and co-cultured with TZM.bl cells as in (A). The percent inhibition of infection (mean±SD, triplicate conditions) is shown for 1 of 4 experiments. A statistically significant difference (p<0.05, two-tailed paired *t*-test) between the enhancement effects on Bal vs RT-SHIV infection is noted by the asterisk. (C) Mature DCs pre-treated with Carraguard (Pre, open squares) were challenged with 3000 TCID_50_ of Bal or 4500 TCID_50_ of RT-SHIV, washed, and co-cultured with TZM.bl cells. Alternatively, mature DCs were pulsed with virus, washed, added to TZM.bl cells and the graded doses of Carraguard added to the co-cultures (Post, filled squares). The percent inhibition (mean±SD, triplicates) are shown from 1 of 4 experiments. A statistically significant difference (p<0.01, two-tailed paired *t*-test) between the pre versus post Carraguard enhancement effects on Bal infection is noted by the asterisk.

Mature DCs readily transmitted both HIV_Bal_ and RT-SHIV to the recipient cells. Significant increases in the levels of infection were observed in the presence of 10 µg/ml of Carraguard (Fig. 2A; *p*<0.05 for 2×10^3^–5×10^4^ TCID_50_/ml of HIV_Bal_ and for 3×10^3^–1.5×10^4^ TCID_50_/ml of RT-SHIV). Using sub-saturating concentrations of virus, significant levels of enhancement were observed over Carraguard concentrations ranging from 2 to 50 µg/ml, with peak levels of enhancement occurring at 6 µg/ml for both viruses (Fig. 2B). The peak enhancement of RT-SHIV infection was significantly higher than that observed for HIV_Bal_ (*p*<0.04). To more closely dissect the mechanism of this enhancement, mature DCs were loaded with virus in the presence (Pre) or absence of Carraguard and then also included Carraguard in the DC/recipient co-cultures (Post; for those DCs not pre-treated with Carraguard). Enhancement of infection by the lower Carraguard doses was observed when it was present either during the virus pulse of the DCs (Pre) or during the DC/recipient cultures (Post) (Fig. 2C). The peak level of enhancement of RT-SHIV infection was not significantly different in either condition (*p*>0.05), although the peak enhancement of HIV_Bal_ infection was significantly greater for the pre-treated condition (*p*<0.01). Since increased conjugate formation between DCs and the recipient cells would enhance virus transmission and subsequent replication [55], the formation of conjugates was monitored in the presence of the low “enhancing” dose of Carraguard. However, the presence of 6 µg/ml of Carraguard, did not affect the percentages of mature DC-CD4 T cell conjugates (14.73%±2.12 vs 13.93%±2.18 for cells cultured in medium vs Carraguard for 24 h).

### Carraguard inhibits infection of immature DCs coincident with DC maturation

Given the unexpected result that low doses of Carraguard increased virus replication facilitated by mature DCs, the impact of Carraguard on infection of immature DCs was investigated. Monitoring immature DC infection in contrast to mature DC-mediated infection is important at several levels. Firstly, immature DCs in healthy non-inflamed mucosa are hypothesized to be one of the first leukocytes contacting HIV [14], [23], [56]. Secondly, the dynamic nature of DC interactions with CD4 T cells provides immature DCs the capacity to be highly efficient at viral transfer at low numbers over long periods of time [10], [19], [57], [58].

**Figure 3:**
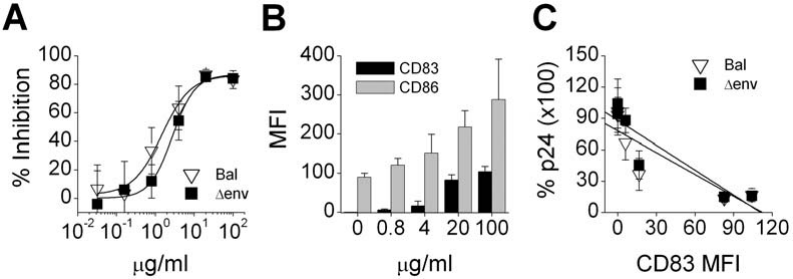
Carraguard inhibits infection in immature DCs coincident with DC maturation. (A) Immature DCs were pre-incubated with graded doses of Carraguard, after which the DCs were challenged with Bal (down triangles) or HIV Δenv pseudotyped with the VSVg envelope (Δenv, filled squares). Cells were harvested 5 d later and stained for (A) HIV capsid p24 protein (mAb KC-57-RD1) or (B) the surface maturation markers CD83 and CD86. (A) Percent inhibition (mean±SD, triplicates) of infection and (B) the MFIs (mean±SD of triplicates) of CD83 (black bar) and CD86 (grey bar) expression (on the entire DC population) are shown for 1 of 4 replicate experiments. CD83 and CD86 up-regulation in response to increasing doses of Carraguard correlate closely (r = 0.99). (C) p24 expression is plotted against CD83, showing the correlation between lower CD83 levels and HIV infection. Comparable results were obtained when comparing CD86 and p24 expression (data not shown).

Carraguard potently inhibited HIV_Bal_ infection of immature DCs in a dose-dependent manner (Fig. 3A, IC_50_ = 1.61 µg/ml±0.21, n = 4), as detected by flow cytometry analysis of HIV p24 expression (since residual Carraguard interferes with the PCR assay). Upon examining the cultures it was apparent that the immature DCs were reacting to Carraguard; the cells rapidly adhered to the plates (within 2 hours) and then cell clustering (as seen with maturing DCs) was more apparent over time. In addition, cytokine profiles of immature DCs treated with increasing doses of Carraguard revealed production of TNF-α at doses greater than 50 µg/ml (data not shown). Flow cytometric analysis of Carraguard-treated DCs revealed a dose-dependent up-regulation of the DC maturation markers CD83 and CD86 (Fig. 3B). Knowing that DC maturation significantly impacts the levels of HIV replication, even when the stimulus is added after virus has been captured by the DCs [59], [60], we wanted to ascertain whether the Carraguard effect was acting at a later stage of the virus life cycle (and not necessarily at the level of virus capture or spread between cells). To do so, immature DCs were infected with VSVg envelope pseudotyped HIV NL43 delta envelope (VSV-HIV Δenv, which will not spread between cells), 16 hours later the cells were washed, and graded doses of Carraguard were added. As with HIV_Bal_ infection, Carraguard blocked VSV-HIV Δenv infection of immature DCs (Fig. 3A; IC_50_ = 1.61 µg/ml±0.21, n = 4 for both viruses). Because Carraguard was added 16 hours after the addition of virus and VSV-HIV Δenv would not spread between cells, the inhibitory capacity of Carraguard could not be associated with initial attachment and fusion.

To further correlate the maturation of immature DCs with inhibition of viral production, the appearance of the HIV p24 antigen was plotted against the mean fluorescence intensity (MFI) of the maturation marker CD83 (Fig. 3C). The appearance of CD83 negatively correlated with the expression of intracellular HIV p24 by DCs infected with either HIV_Bal_ or VSV-HIV Δenv (*r* = −0.86, *p*<0.03 and *r* = −0.91, *p*<0.02 for HIV_Bal_ and VSV-HIV Δenv, respectively; n = 4 for both viruses). Thus, the ability of the compound to inhibit viral replication in this setting appeared to be due to its ability to induce DC maturation.

### The NNRTI MIV-150 overcomes Carraguard-mediated enhancement of infection

Preliminary *in vitro* studies examining the NNRTI MIV-150 (50 µM) and Carraguard in a combination formulation designated PC-815 revealed that combining these agents results in improved inhibitory effects against R5 clinical HIV-1 isolates and an HIV-2 isolate [37]. Knowing that blockade of DC-driven infection can require greater anti-viral drug potency to limit infection than is required in other *in vitro* assays [61], [62], we sought to determine the efficacy of a high dose (500 µM) MIV-150/Carraguard combination formulation designated PC-817 in our DC systems and *in vivo*.

**Figure 4:**
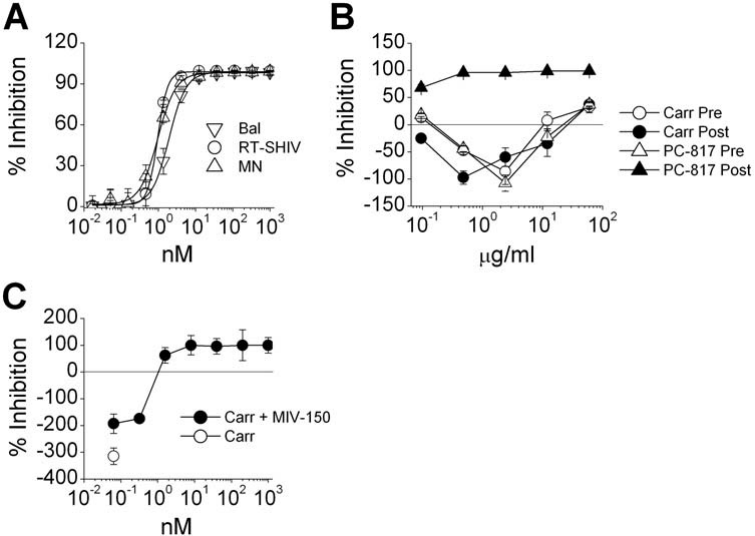
MIV-150 overrides the augmentation of DC-driven infection by Carraguard. (A) TZM.bl cells were pre-exposed to varying doses of MIV-150, challenged with the indicated viruses. Mean percent inhibition (±SD, triplicates) is shown from 1 of 3 experiments. (B) Mature DCs pre-treated with Carraguard (Carr Pre, open circles) or PC-817 (PC-817 Pre, open triangles; 33.3 nM MIV-150 per 2 µg/ml Carraguard) were pulsed with RT-SHIV, washed, and co-cultured with TZM.bl cells. Alternatively, mature DCs were pulsed with virus, washed, added to TZM.bl cells and the graded doses of Carraguard (Carr Post, filled circles) or PC-817 (PC-817 Post, filled triangles) added to the co-cultures. The concentrations indicated on the X axis indicate the Carraguard concentrations for each formulation. The percents of inhibition (mean±SD, triplicates) are shown for 1 of 3 similar experiments. (C) Mature DCs were treated with 2 µg/ml of Carraguard, pulsed with RT-SHIV, washed, and then cultured with TZM.bl cells in the presence (Carr+MIV-150) or absence (Carr) of varying doses of MIV-150 (nM). Percent inhibition of infection (mean±SD, triplicates) are shown for 1 of 3 identical experiments.

Unlike Carraguard, MIV-150 potently inhibited infection of TZM.bl cells with HIV_Bal_, RT-SHIV, and HIV_MN_ at concentrations below 3 nM (HIV_Bal_ IC_50_ = 2.94 nM±1.20; RT-SHIV IC_50_ = 0.99 nM±0.10; HIV_MN_ IC_50_ = 1.03 nM±0.27; n = 3 for each virus) (Fig. 4A). We attempted synergy analysis using the Chou-Talay algorithm as previously described [63], although both HIV_Bal_ and RT-SHIV inhibition/enhancement curves did not satisfy the basic assumptions of this type of analyses (i.e., the shape and potency of the Carraguard curves were different to the typical sigmoidal curve for MIV-150). Therefore, we restricted our studies to the ability of MIV-150 to overcome the enhancement observed in mature DC-driven RT-SHIV infection, the experimental system that was most significantly enhanced by Carraguard. Pre-treatment of DCs with either Carraguard or PC-817 and post-treatment with Carraguard resulted in comparable levels of enhancement of RT-SHIV infection, while addition of PC-817 to the DC/recipient mixtures (post-treatment) effectively inhibited virus replication (Fig. 4B). Thus, the presence of MIV-150 overcame the enhancing effects of Carraguard. To more closely examine the amounts of MIV-150 needed to overcome the Carraguard enhancement of infection, titrated doses of MIV-150 were added to mature DC/recipient mixtures in the presence of 2 µg/ml Carraguard. Less than 1 nM of MIV-150 partially reversed the enhancement by Carraguard, with complete inhibition of virus replication being observed in the presence of as little as 8 nM of MIV-150 (Fig. 4C, *p*<0.001).

### Carraguard-based gels inhibit immunodeficiency virus transmission *in vivo*

**Figure 5:**
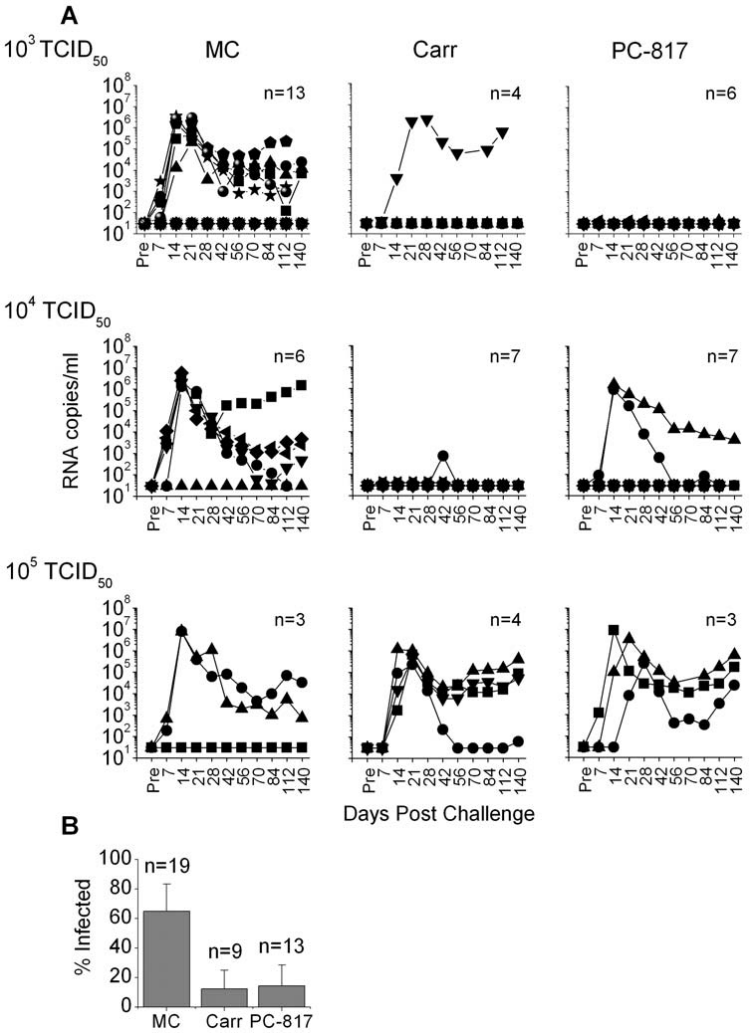
Carraguard-based gels inhibit vaginal infection in macaques. Depo-Provera-treated animals were treated with 3 ml of MC, Carraguard (Carr), or PC-817 30 min prior to challenge with 1 ml of or 10^3^–10^5^ TCID_50_ RT-SHIV. (A) Plasma viral loads were quantified by PCR and SIV gag RNA copies per ml of plasma are shown for each animal over time. The numbers of animals in the respective groups are indicated in each panel. Each symbol denotes a different animal (Table 1). (B) The frequencies (percentage of infected animals, mean±SEM) of infection in the MC, Carraguard, and PC-817-treated groups challenged with 10^3^ and 10^4^ TCID_50_ are plotted. Carraguard (p<0.02) and PC-817 (p<0.03) significantly reduced the frequency of immunodeficiency virus infection compared to the MC-treated placebo group.

Concurrent with the *in vitro* research, *in vivo* studies were carried out in order to compare Carraguard and PC-817 for their ability to prevent vaginal RT-SHIV infection in macaques. Depo-Provera-treated macaques had 3 ml of the indicated gels applied vaginally and then varying doses of RT-SHIV were applied 30 min later. Doses of >3000 TCID_50_ of SIVmac239 (parental strain of RT-SHIV) were shown to be relatively high based on frequency of animal infections [64] and 300 TCID_50_ of SHIV162P3 is commonly used in testing other microbicide strategies [65], [66]. Therefore, we compared a similar low end dose of 10^3^ TCID_50_ of RT-SHIV to two higher doses (10^4^ and 10^5^ TCID_50_), to more rigorously test the Carraguard-based gels. A 2.5% MC gel was used as the placebo to parallel the products used in the Phase III clinical trial [8]. Vaginal challenge with RT-SHIV infected animals in a dose-dependent manner, resulting in mean peak viremias of approximately 7.8×10^6^, 2.8×10^6^, and 1.5×10^6^ SIV RNA copies/ml after challenge with 10^5^, 10^4^, and 10^3^ TCID_50_, respectively (66.7%, 83.3%, and 46% infection in the MC-treated groups, Fig. 5). Analysis of the average area under the curve of the viral loads in the MC control groups, confirmed the correlation between the virus challenge dose and virus levels over time (weeks 1 to 16, r^2^ = 0.97).

Both Carraguard and PC-817 protected against vaginal RT-SHIV infection and this was dependent on the dose of challenge inoculum used (Fig. 5A). Neither gel protected against the 10^5^ TCID_50_ dose of RT-SHIV, although the peak viral loads were delayed 1–2 weeks in 3 of the 4 Carraguard-treated animals and 2 of the 3 PC-817 treated animals compared to the infected MC-treated animals (Fig. 5A, bottom row). However, Carraguard and PC-817 reduced RT-SHIV infection at both the 10^4^ and 10^3^ TCID_50_ challenge doses (Fig. 5A, middle and top rows, respectively). The 2 PC-817-treated animals infected after the 10^4^ TCID_50_ challenge showed typical plasma viremia peaking after 14 days, while the 1 Carraguard-treated animal infected after the 10^3^ TCID_50_ challenge showed a delayed peak viremia compared to the MC-treated animals. Independent of the virus challenge dose and gel exposure, all animals that became infected with RT-SHIV (repeated positive samples) exhibited co-culture positivity and developed SIV-specific Ab and cellular responses, as expected (Table 1). One Carraguard-treated animal challenged with 10^4^ TCID_50_ of RT-SHIV showed one low positive reading of <1000 copies/ml, but it was negative at all other time points. This animal did not appear to have developed adaptive SIV-specific responses that might have controlled infection (Table 1), suggesting that either innate responses controlled infection, that this was a false positive RNA result, or this was evidence of an abortive infection [67]. This was supported by the lack of the appearance of virus in the plasma after depletion of CD8 cells (for 2–3 weeks during which time increases in plasma virus levels were observed in positive control animals) using the mAb cM-T807 [68] (data not shown).

**Table 1:** Infection and immune status of RT-SHIV-challenged macaques.

Gel	Virus	Animal #	Infection	Culture$	Ab	IFNγ	CD4 cells/µlˆ	
							Pre	112 d post
**MC**	**10^3^ RT-SHIV**	FI80	+	+	+	+	1555	715
		FI81	+	+	+	+	3204	1618
		FI82	+	+	+	+	1679	1478
		DE38&	−	−	−	+	894	1283
		GF15	−	−	−	−	556	565
		GF16	−	−	−	−	1657	896
		GJ43	+	+	+	+	1177	528
		GJ51	+	+	+	+	1543	966
		GK06	−	−	−	−	2346	1070
		GK05	−	−	−	−	596	877
		GF13	−	−	−	−	644	908
		GJ29	−	−	−	−	883	823
		GJ28	+	+	+	+	413	605
	**10^4^ RT-SHIV**	GF23	+	+	+	−	912	879
		FI76	+	+	−	+	1498	1019
		GF17	−	−	−	−	1016	1377
		GF18	+	+	+	+	1941	2732
		FI72	+	+	+	+	1501	616
		FI74	+	+	+	+	1353	1178
	**10^5^ RT-SHIV**	FI76	−	−	−	−	1029	1617
		FI77	+	+	−	+	1061	ND
		FI78	+	+	+	+	976	1135
**Carraguard**	**10^3^ RT-SHIV**	FI72	−	−	−	−	2381	3097
		FI74	−	−	−	−	2652	3539
		FI79	−	−	−	−	2457	2229
		GJ38	+	+	+	−	303	420
	**10^4^ RT-SHIV**	GF13	−	−	−	−	1161	2346
		GF14	−*	−	−	−	404	1064
		GF15	−	−	−	−	663	882
		GF16	−	−	−	−	1141	2175
		FI72	−	−	−	−	1719	1973
		FI74	−	−	−	−	2738	1981
		FI79	−	−	−	−	1920	1306
	**10^5^ RT-SHIV**	FI70	+	+	+	+	821	412
		FI71	+	+	−	+	1042	1425
		FI73	+	+	+	+	2984	1467
		FI75	+	+	+	+	1712	805

Gel	Virus	Animal #	Infection	Culture$	Ab	IFNγ	CD4 cells/µl	
							Pre	112 dˆ post
**PC-817**	**10^3^ RT-SHIV**	FI83	−	−	−	−	1337	1454
		FI84	−	−	−	−	1806	3142
		FI85	−	−	−	−	1531	1119
		FI88	−	−	−	−	1107	1363
		GJ34	−	−	−	−	956	1029
		GJ41	−	−	−	−	981	972
	**10^4^ RT-SHIV**	GF07	−	−	−	−	1744	2460
		GF08	+	+	+	+	965	1266
		GF12	+	+	+	+	1673	1895
		FI83	−	−	−	−	1920	933
		FI84	−	−	−	−	2194	1428
		FI85	−	−	−	−	935	1312
		FI88	−	−	−	−	1297	1381
	**10^5^ RT-SHIV**	FI86	+	+	+	+	1018	1092
		FI87	+	+	−	+	1429	568
		FI89	+	+	+	+	2092	ND
**MIV-150 1X** %	**10^3^ RT-SHIV**	DK96	+	+	+	+	1106	914
		DE54	−	−	−	−	1241	1588
		GF13	−	−	−	−	1244	889
		GF15	+	+	+	+	507	171
		GF17	−	−	−	−	560	478
		GJ31	−	−	−	−	403	383
		GK01	+	+	+	+	1744	1398
**MIV-150 1X** %	**10^4^ RT-SHIV**	DE38	+	+	+	+	1092	722
		DE54	+	+	−	−	1549	467
**MIV-150 3X** %	**10^4^ RT-SHIV**	GF21	+	+	+	+	881	926
		FI83	+	+	+	+	1095	632
		FI85	+	+	+	−	1088	1443
		GF20	−	−	−	−	505	999
		FI79	-/+@	−	−	−	1549	1007
		FI84	-/+@	−	−	−	1327	1443
		FI88	-/+@	−	−	−	738	784

ˆ CD4 counts taken on day 84 for DE38, day 189 for FI76, FI78, FI70, FI71, FI73, and FI75, and day 196 for FI86 and FI87.

$ Co-cultures of PBMCs with 174×CEM cells were scored as positive by microscopic examination of syncytia formation and in some instances verified by PCR for SIV gag.

& DE38 had two timepoints of positive IFNγ responses at weeks 8 and 10 post challenge.

* GF14 - average 715 copies/ml on day 42 post challenge, but the animal remained below the level of detection at all other time points.

% 500 µM of MIV-150 in MC administered once 30 min prior to challenge (1X) or 24 h before, 30 min before, and 30 min after virus challenge (3X).

@ These animals exhibited low-level viremia but ultimately controlled infection to undetectable levels even after CD8 depletion.

Ab positivity was defined as having at least two positive OD values above baseline at 4 weeks post challenge and IFNγ positivity was defined as having at least 50 SIV-specific IFNγ SFCs per 10^6^ PBMCs on more than one time point post challenge.

ND, not determined.

Due to the small sample size of each TCID_50_ challenge group, statistical analyses were performed on the combined data from the 10^3^ and 10^4^ TCID_50_ groups where there was at least some protective effect of the gels (Fig. 5B). Both Carraguard and PC-817 significantly inhibited infection (p<0.02 and p<0.03, respectively), reducing the frequency of infection to at least 75% of that seen in the MC-treated animals.

### Activity of topically applied MIV-150 against RT-SHIV *in vivo*

**Figure 6:**
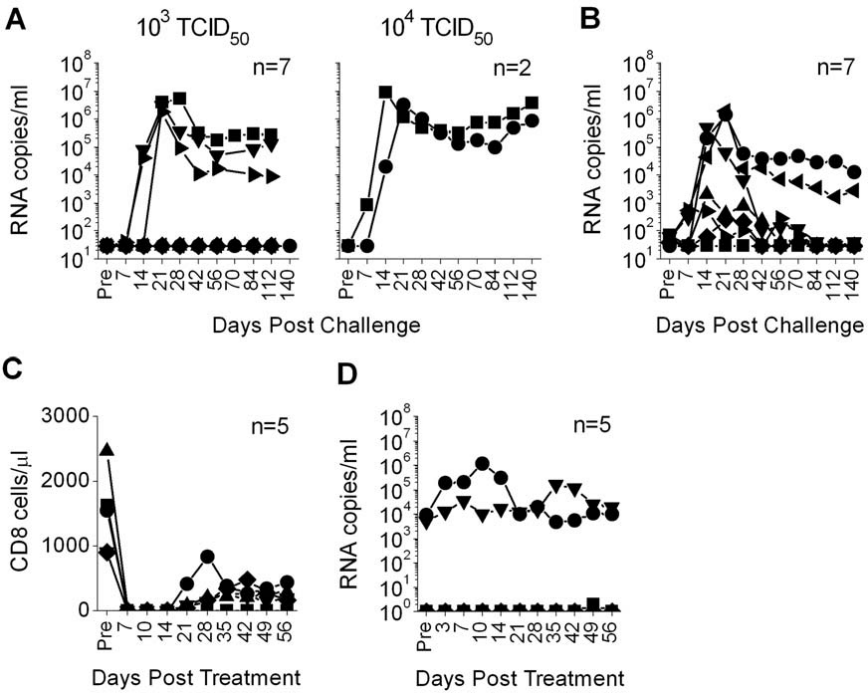
*In vivo* activity of MIV-150-containing MC gels. Depo-Provera-treated animals were treated with (A) 3 ml of MC containing 500 µM MIV-150 30 min prior to vaginal challenge with 10^3^ or 10^4^ TCID_50_ of RT-SHIV or (B) 3 ml of MC containing 500 µM MIV-150 24 h before, 30 min before, and 24 h after vaginal challenge with 10^4^ TCID_50_ of RT-SHIV. Plasma viral loads over time are shown for the indicated numbers of animals in each group. One year after challenge the 3 animals with the low-level initial infection (now with undetectable virus) and two of the normally infected animals were treated with the anti-CD8 mAb to deplete CD8 cells. (C) Effective depletion of CD8 cells was verified by flow cytometry and the CD8 cells per µl of blood are shown for each animal. (D) Analysis of the plasma virus loads before during and after CD8 depletion, revealed no rebound in virus levels in the 3 animals with the unusual acute low-level infection. Each symbol denotes a different animal that are detailed in Table 1.

Since PC-817 did not appear to exhibit any additional activity against vaginal RT-SHIV challenge above that seen with Carraguard, we examined the ability of MIV-150 in MC to limit vaginal spread. As predicted by the PC-817 data (Fig. 5), a single dose of 500 µM of MIV-150 in MC given 30 min prior to vaginal challenge with 10^3^ or 10^4^ TCID_50_ of RT-SHIV did not prevent infection, although the peak viremia was delayed by 1–2 weeks in all animals infected after challenge with 10^3^ TCID_50_ and in one of the animals receiving the higher inoculum (Fig. 6A). However, repeated doses of 500 µM of MIV-150 (1.5 mM total) in MC given 24 h before, 30 min before, and 24 h after challenge did reduce RT-SHIV infection, delaying the peak viremia in 2 animals (Fig. 6B). Compared to the MC control where 5 of 6 (∼83%) animals challenged with 10^4^ TCID_50_ became infected (Fig. 5A), only 3 of 7 (∼43%) animals receiving the 3 doses of MIV-150 in MC got infected and exhibited typical plasma viremias; MIV-150 reduced the frequency of normal infections by ∼49%. Surprisingly, 3 other animals were virus positive on repeated occasions after challenge, but their peak viremias were 3–4 logs lower than the placebo group, with virus ultimately becoming undetectable after 1.5–3 months. Unlike typically infected animals, CD8 depletion of these 3 animals with the abnormally low infection did not result in any rebound of plasma virus levels (Fig. 6C and D). Just like the one uninfected animal, these animals did not develop detectable SIV-specific Ab or T cell responses, which are observed in most infected animals (Table 1). Thus, topically applied MIV-150 can limit vaginal RT-SHIV infection.

## Discussion

*In vitro* predictive indicators of potential microbicides are needed prior to any animal efficacy testing. Although which tests can predict *in vivo* efficacy is of much debate. The study herein, presents an extreme example where most *in vitro* results were discordant with those observed *in vivo* at several levels. For instance, based on *in vitro* data alone, there is little supporting evidence that Carraguard would be protective *in vivo*. Rather, significant levels of enhancement were observed *in vitro* with our challenge virus RT-SHIV when Carraguard levels were below 10 µg/ml in cell-free infections of cell lines and activated PBMCs and also in cell-associated assays where mature DCs were used to transfer to recipient cells. These enhancing effects were more pronounced than has been reported previously [37], probably due to the lower doses of gels employed herein, as well as the use of mature DC assays and/or differing viral strains. The only exception, in accord with the 10^3^ and 10^4^ TCID_50_ *in vivo* results, was the potent inhibition of immature DC infection. The latter is likely the result of indirect effects, as Carraguard also matured the DCs, and DC maturation is associated with the reduced capacity of viral production [16], [69]–[72], even when DCs receive the maturation stimulus after virus exposure [59], [60].

Considerable evidence has demonstrated that polyanions like Carraguard are significantly more effective against the positively charged X4 viruses [24]–[29]. There have also been some reports of polyanions enhancing infections on the basis of interactions with the V1/V2 regions of envelope [26], [27], [73]. Herein, it is apparent that the level of enhancement is also dependent on the R5 isolate used, being most pronounced with RT-SHIV in the mature DC/recipient co-cultures. The mechanism of *in vitro* enhancement remains unclear and cannot be attributed to the two main mechanisms needed for DC viral transfer: increased capture of virus by the DCs or increased DC-T cell conjugate formation. In contrast to the polyanion attachment inhibitor class, the NNRTI MIV-150 consistently was not only potent, but also very effective against the virus isolates used with this study. Importantly, MIV-150 also overcame the unsuspected enhancing effects of Carraguard in the mature DC/recipient mixtures. This supports the initial report showing the potent effects of Carraguard and MIV-150 *in vitro* against several R5 Clade C clinical isolates [37].

Based on these *in vitro* findings we would have predicted that Carraguard would exhibit limited efficacy in preventing R5 RT-SHIV infection *in vivo*, while the addition of MIV-150 to Carraguard would render PC-817 considerably more effective. This was, however, not the case and the *in vivo* data disagreed with most of the *in vitro* data at three levels. Firstly, Carraguard did not enhance infection *in vivo*. Secondly, Carraguard proved to be effective against relatively high dose challenges (10^3^ and 10^4^ TCID_50_) of RT-SHIV. Thirdly, due to the efficiency of Carraguard it was not possible to demonstrate any difference between Carraguard and PC-817, with them both reducing infection by at least 75% of that seen in MC placebo controls. This is not the first observation of discrepancies between *in vitro* and *in vivo* data. Several *in vivo* studies demonstrated that the level of the compound needed to prevent infection *in vivo* is several orders of magnitude higher than that seen *in vitro* [65], [74]–[76]. Although in all cases, investigators did not observe enhancement *in vitro* of their test compound prior to *in vivo* testing. Thus, the study herein presents a novel comparison where enhancement *in vitro* contradicted effective inhibition *in vivo*.

Understanding which variable(s) were dominant *in vivo*, may aid in the future design of *in vitro* assays to more consistently predict the potential outcomes in animal models and ultimately in humans. As in previous microbicide challenges in macaques [4], [65], [66], [76], [77], there is an inordinately large amount of compound being used topically relative to the test levels *in vitro*. The *in vivo* dose of Carraguard (30 mg/ml) is at least 300 fold higher than the maximum levels that can be used *in vitro* and more than 3000 fold higher than the doses at which *in vitro* enhancement was observed. This is largely due to the viscosity of the 30 mg/ml solution, which is necessary for it to remain in place *in vivo*, but which interferes with the *in vitro* assays. Complementing our immature DC infection data, *in vitro* studies using tissue explants demonstrated that Carraguard was able to reduce R5 HIV_Bal_ infection by 50%, even when it was used at only 3 mg/ml (10 fold less than the recommended *in vivo* dose) [78]. It is likely that Carraguard-based gels coat the epithelial surfaces providing a primary barrier against HIV entering the tissues and reaching the underlying leukocytes that would amplify infection. Only rare DC processes extending to the epithelial surface or breaks in the tissue caused by trauma or other infections would afford the gels direct access to the underlying DCs. Since immature DCs dominate in healthy epithelia, our data indicate that if Carraguard was to reach the cells it would limit infection of the immature DCs under these circumstances. As such, it is likely that the gel rarely encounters mature DCs and T cells in which the enhancing effects were observed *in vitro*. Importantly, there was no evidence of enhancement of infection in macaques when the gels were applied atraumatically 30 min prior to challenge. Having only ∼50% infection frequency in the control 10^3^ TCID_50_ challenge group would have allowed the detection of any significant adverse enhancing effects of the gels if they had occurred. Analysis of macaque vaginal swabs within the first days after a single application of PC-817 (versus MC or nothing, 4 animals per group) revealed no change in the levels of cytokines or chemokines (Melissa Robbiani and Rachel Singer, unpublished observations). This suggests a lack of immunomodulation *in vivo* as reported recently in women [79]. There was no evidence of inflammation or enhancement of infection in the recently completed Phase III trial of Carraguard [8]. Furthermore, use of Carraguard 2–3 times a week has been reported to be acceptable and safe, affording no differences to the placebo-using couples [80] and daily use of Carraguard for 7–14 d was found to be safe in HIV-infected men and women [81].

Clearly, the outcome of the Phase III clinical trial is in opposition to the *in vivo* macaque data presented within, highlighting that challenges still lie ahead in the study design of animal models predicting efficacy in clinical trials. For instance, there are several variables that need to be considered in future animal trials. Firstly, the placebo control gels need to match the inherent rheological properties of the gel to be tested. The MC placebo used herein did not exactly match rheological properties of Carraguard, however MC was used in parallel in Phase III clinical trials and therefore cannot explain the contrasting observations in the animal studies presented herein. Secondly, the means by which the virus is introduced is also of importance, as the atraumatic application of cell-free virus in the macaque model diverges from that occurring in heterosexual HIV transmission in humans. Key variables in the latter situation are physical and sometimes traumatic (e.g., cultural practices such as dry sex) introduction of virus in the form of either a cell-associated or cell-free viral inocula in the presence of seminal fluid. The presence of seminal fluid as an additional variable may actually increase the effective viral inocula present, due to the presence of semen-derived amyloid fibrils [82]. However, Carraguard-based formulations show no limitations in activity in the presence of seminal fluid *in vitro* [37].

Inclusion of an additional agent that broadens the activity of a formulation (overcoming any potential limitations of the gel) is highly advantageous. To this end, extremely low doses of MIV-150 effectively inhibited infection and overcame any enhancing effects of Carraguard *in vitro*, suggesting that this would increase protection *in vivo* when comparing Carraguard versus PC-817. Unfortunately, improved efficacy of PC-817 over Carraguard *in vivo* could not be demonstrated, due to the potency of Carraguard under these conditions, probably as a result of Carraguard's strong barrier effect. Supporting this, we observed that a single 500 µM dose of MIV-150 in MC given 30 min prior to challenge was unable to protect against vaginal infection. However, the *in vivo* activity of topically applied MIV-150 was verified by the fact that repeated doses of MIV-150 in MC reduced vaginal infection. This possibly reflects the dosing and/or timing requirements for the activity of MIV-150 to be evident. Since MIV-150 has both anti-viral and virucidal activities, the need for higher doses could indicate the need for more drug to be absorbed into the tissues and/or that the virucidal effect is needed to limit spread, since more MIV-150 was needed for virucidal effects *in vitro* [37]. Ongoing macaque studies are exploring how MIV-150 is acting *in vivo*. These findings set the stage for future studies examining the repeated (e.g., daily) application of gels that would allow women to use them independent of coitus and macaque studies using Carraguard-based gels (with and without MIV-150), are underway. Also, the topical activity of the NNRTI MIV-150 indicates promise for alternative strategies employing anti-viral drug-containing microbicide vaginal rings.

These studies provide direct evidence that Carraguard-based gels can afford protection against immunodeficiency virus infection when they are applied before atraumatic cell-free virus exposure in the absence of seminal fluid. However, the lack of protection within the recent Carraguard clinical trial emphasizes how other variables in humans need to be addressed in future study design using macaques in microbicide testing. These include the viral inocula, the presence of seminal fluid, the challenge virus used, single versus multiple virus applications and inclusion of mechanical disruption that mimics coitus. Unfortunately, not all variables can be addressed in animal models. For instance, additional post-hoc survival analyses in the Phase III clinical trial of Carraguard based on behavior, other risk factors, and adherence levels revealed that HIV incidence continued to be lower in the Carraguard group among the women who used the gel during 100% of sex acts, although this also did not reach statistical significance (Robin Maguire, personal communication). Importantly, adherence to using the gel was a significant problem and it is not clear when the supposedly compliant women actually used the gel relative to sexual intercourse. It is quite likely that the window of opportunity for a gel to be effective when used as a single application will be narrower than that for repeated (e.g., daily) use, thereby further undermining the efficacy of a single dose product. In summary, the data herein demonstrate the efficacy of Carraguard-based gels at preventing vaginal infection in a controlled environment. Moreover, we provide the first evidence that topically applied MIV-150 can restrict vaginal RT-SHIV infection. Together, this sets the stage for future research on anti-HIV strategies (repeated gel applications, or long term drug release from vaginal rings) that would afford greater activity against HIV infection.
